# Periosteal Stripping and Periosteal Division for Leg Length Discrepancy After Proximal Femoral Intertrochanteric Osteotomy for Perthes Disease

**DOI:** 10.7759/cureus.70953

**Published:** 2024-10-06

**Authors:** Ayana Kitta, Takashi Saisu, Jun Kakizaki, Yasuhiro Oikawa, Yuko Segawa, Ken Okazaki

**Affiliations:** 1 Division of Orthopedic Surgery, Tokyo Women's Medical University Yachiyo Medical Center, Chiba, JPN; 2 Division of Orthopedics, Chiba Child and Adult Orthopedic Clinic, Chiba, JPN; 3 Division of Orthopedic Surgery, Chiba Children's Hospital, Chiba, JPN; 4 Division of Orthopedic Surgery, Chiba Children’s Hospital, Chiba, JPN; 5 Division of Orthopedic Surgery, Tokyo Medical and Dental university, Tokyo, JPN; 6 Division of Orthopedic Surgery, Tokyo Women’s Medical University, Tokyo, JPN

**Keywords:** growth plate, leg length correction, leg length discrepancy, perthes disease, the periosteal stripping and periosteal division

## Abstract

Background and objective

Periosteal stripping and periosteal division (PSPD) can help promote the growth of the long bone in children with leg length discrepancy (LLD). We performed PSPD in a cohort of patients when LLD was observed at the time of implant removal surgery after proximal femoral osteotomy for Perthes disease. This study aimed to clarify the efficacy and safety of PSPD for acquired LLD related to Perthes disease.

Methods

This retrospective study enrolled 10 patients treated with PSPD and six controls who declined PSPD for LLD associated with Perthes disease. The lengths of the femur, tibia, and entire leg were measured in the full-length standing radiographs at baseline and final follow-up. The baseline was defined as the time of the last preoperative observation. LLD and changes in LLD (ΔLLD) were measured. The correlation of ΔLLD with age at the time of surgery, follow-up period, and extent of PSPD was investigated.

Results

In the PSPD group, the mean age of the patients was 9.4 years and the mean LLD at baseline was 20.5 ± 4.6 mm, while it was 10.2 years and 11.5 ± 10.0 mm in the control group. With a mean follow-up period of 4.3 years, the PSPD group showed a mean ΔLLD decrease of 13.9 mm, which was significantly greater than that of the control group at 3.2 mm with a mean follow-up period of 5.4 years. Logistic regression analysis revealed that age at the time of surgery was a significant factor for obtaining >10 mm ΔLLD with PSPD and the cutoff value by the receiver operating characteristic curve was 9.6 years (sensitivity: 0.83; specificity: 0.83).

Conclusions

PSPD seems to be a safe and effective surgical option for LLD associated with Perthes disease. The age at the time of surgery negatively correlated with the amount of LLD correction. Obtaining >10 mm LLD correction is more likely if the patients are <10 years of age.

## Introduction

Leg length discrepancy (LLD) can cause various clinical problems, such as scoliosis, gait abnormalities, and the early onset of osteoarthritis [1,2]. The causes of LLD include lower limb hypoplasia, unilateral hypertrophy due to neoplastic lesions, growth disturbances due to trauma or infection, slipped capital femoral epiphysis (SCFE), and Perthes disease [1,3,4]. Perthes disease patients can suffer from LLD reaching up to 7 cm [5-7]. The causes of LLD associated with Perthes disease include the spread of the femoral head lesion [8-10], premature closure of the proximal femoral growth plate [11-15], disuse bone atrophy due to orthotic unloading [16], and proximal femoral intertrochanteric osteotomy [8,16,17].

Surgical treatments for LLD include acute shortening, acute or gradual bone lengthening, growth plate suppression, and periosteal stripping and periosteal division (PSPD) [18]. Acute shortening may cause a transient limp due to relative muscle length shortening, and acute lengthening may lead to traction neuropathy [19]. Gradual bone lengthening with external fixation is associated with some risk of complications, including knee and ankle joint contracture due to soft tissue elongation, traction neuropathy, and pin site infection. Additionally, the potential psychological impact of long-term treatment needs to be considered [20]. Growth plate suppression targets the growth plate of the longer side of the leg. Temporary growth suppression using the eight-plates has been widely used recently due to its simplicity and low invasiveness [21]. Nevertheless, several complications, such as loosening or breaking of the screws, premature closure of the growth plate [22], and changes in proximal tibial bone morphology [23], have been reported. The suppression effect is difficult to predict.

PSPD is a technique for promoting the growth of the long bone, as reported by Limpaphayom et al. in 2011 [18]. They reported 11 cases with a mean preoperative LLD of 60 ± 38 mm and a mean age of 9.0 ± 2.5 years. PSPD was reportedly performed on the entire length, and correction of LLD was achieved in eight of 11 patients in an average of 25 ± 17.2 months and was maintained throughout the follow-up period [18]. PSPD is less burdensome to patients as it involves only one surgery; however, predicting the amount of leg length correction is difficult in PSPD. Moreover, there is scarce data on the clinical results of PSPD in the literature.

At our hospital, we offer PSPD for cases with LLD at the time of implant removal surgery after proximal femoral intertrochanteric osteotomy for Perthes disease, if the patients and their families so desire. The present study aimed to clarify the efficacy and safety of PSPD for acquired LLD related to Perthes disease. Additionally, we sought to examine the correlations between the leg length correction effect, age at the time of surgery, and the extent of surgery to predict the amount of leg length correction with PSPD. We hypothesized that the younger the patient at the time of surgery and the larger the area of surgery, the greater the leg length correction effect.

This article was previously presented as a meeting abstract at the Annual Meeting of the Japanese Pediatric Orthopaedic Association on December 18, 2018. It was also posted on the preprint platform ResearchSquare on January 3, 2023. The present study was registered in the original registration system at Chiba Children’s Hospital (registration no: 2021-074).

## Materials and methods

Study design and setting

In this retrospective study, we followed up on patients treated for Perthes disease to evaluate outcomes. The study was conducted at the Chiba Children’s Hospital, Japan, under the supervision of Dr. Takashi Saisu.

Patients

From August 2012 to August 2020, we performed PSPD in 10 children with LLD related to Perthes disease. The inclusion criteria were as follows: 1) patients with unilateral Perthes disease; 2) LLD of >20 mm before implant removal surgery after proximal femoral intertrochanteric osteotomy or LLD of ≥20 mm at the end of growth based on the clinical course of the patient as predicted by the attending physician; 3) the patient was at the age when the growth plate is still present and PSPD is expected to promote bone overgrowth; 4) the parent and guardian were aware of the surgical risks and benefits and provided consent; 5) radiographs of the entire leg were taken before and after the surgery; and 6) patients were followed up for at least one year after PSPD. The exclusion criteria were as follows: 1) patients with infection of the surgical sites; 2) nonunion of the osteotomy; 3) neurological disorders; and 4) skeletal dysplasia.

Controls

During the study period, 10 cases met the inclusion criteria. During the same period, 12 patients with unilateral Perthes disease underwent proximal femoral intertrochanteric osteotomy followed by implant removal surgery without PSPD. To clarify the natural course of LLD after the proximal femoral osteotomy, the patients who did not undergo PSPD were regarded as the control group. The inclusion criteria of the control group were as follows: radiographs of the entire leg were taken before and after surgery and the patients were followed up for at least one year after surgery. Of these, five cases without preoperative LLD measurement and one case without postoperative LLD measurement were excluded; the remaining six cases met the inclusion criteria.

Surgical procedures

**Figure 1:**
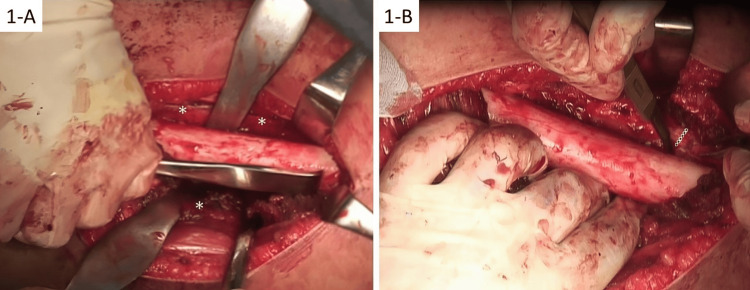
Intraoperative photographs during PSPD A. Periosteal stripping of the femur. A longitudinal incision is made on the periosteum. Arrowheads point to the periosteum. B. Periosteal division. A circular incision is made on the periosteum. The dotted line shows the incision on the periosteum PSPD: periosteal stripping and periosteal division

PSPD was performed as detailed in a previous study [18]. The patient was placed in a lateral position, and the location of the epiphyseal line was confirmed by fluoroscopy. A longitudinal skin incision was made laterally on the femur, anteromedially on the tibia, and laterally on the fibula. A longitudinal incision was made on the periosteum, and the periosteum was dissected circumferentially (Figure 1A). A circular incision was made in the periosteum at the proximal, middle, and distal parts of the diaphysis of the femur (Figure 1B). The same technique was used for the tibia and fibula. A drainage tube was placed on the treated bone to prevent compartment syndrome.

Radiological assessments

The entire leg length was defined as the distance from the center of the hip joint to the center of the ankle joint. The femoral length was defined as the distance from the center of the hip joint to the center of the knee joint. The tibial length was defined as the distance from the center of the knee joint to the center of the ankle joint. The center of the joint refers to both the horizontal and vertical widths of the joint gap. The distances (mm) were measured by PACS in a full-length standing frontal radiographic view of the entire leg. The length difference between the healthy and affected sides was defined as LLD (mm), and ΔLLD (mm) was defined as the value obtained by subtracting the LLD at the last observation from the preoperative LLD.

Statistical analysis

As part of the statistical assessment, preoperative and postoperative LLD were analyzed by performing the Wilcoxon-signed rank test, and the difference in the mean LLD between groups was analyzed with the Mann-Whitney U test. The Pearson’s correlation coefficient was calculated between ΔLLD and age at the time of surgery or follow-up period. The Spearman’s correlation coefficient was calculated between the ΔLLD and the extent of PSPD. Logistic regression analysis was performed to determine whether the ΔLLD effect of ≥10 mm could be obtained using age at the time of surgery as a predictive variable. A p-value <0.05 was considered statistically significant. Statistical analyses were performed using JMP Pro ver. 15 (SAS Institute Inc., Cary, NC).

## Results

The demographic data of the cohort are shown in Table 1. The mean age at the time of PSPD was 9.4 years, and the mean follow-up period was 4.3 years. PSPD was performed on the proximal 1/2 of the femur in five patients, proximal 2/3 of the femur in four patients, and the entire length of the femur and lower leg in one patient. The mean age and follow-up period in the control group were 10.2 and 5.4 years, respectively. All patients were male.

**Table 1:** Demographic data of the patients LLD: leg length discrepancy; Prox.: proximal; PSPD: periosteal stripping and periosteal division

PSPD							
Case	Age at operation, years	Extent of PSPD, mm	Preoperative LLD, mm	Follow-up LLD, mm	ΔLLD, mm	Age at follow-up, years	Follow-up period, months
P1	7	Prox. femur 1/2	18	−2	20	16	108
P2	8	Prox. femur 1/2	27	9	18	11	39
P3	10	Prox. femur 1/2	17	8	9	14	26
P4	11	Prox. femur 1/2	28	22	6	17	73
P5	11	Prox. femur 1/2	21	17	4	14	41
P6	6	Prox. femur 2/3	16	−4	20	9	40
P7	7	Prox. femur 2/3	14	−10	24	9	27
P8	9	Prox. femur 2/3	20	10	10	12	37
P9	13	Prox. femur 2/3	20	14	6	17	55
P10	12	Femur, tibia, fibula	24	2	22	19	90
Controls							
Case	Age at implant removal, years		Preoperative LLD, mm	Follow-up LLD, mm	ΔLLD, mm	Age at follow-up, years	Follow-up period, months
C1	7		10	−4	14	10	45
C2	8		12	11	1	14	80
C3	9		5	12	−7	19	117
C4	9		6	−6	12	17	101
C5	13		31	32	−1	16	34
C6	15		5	5	0	16	15

**Figure 2:**
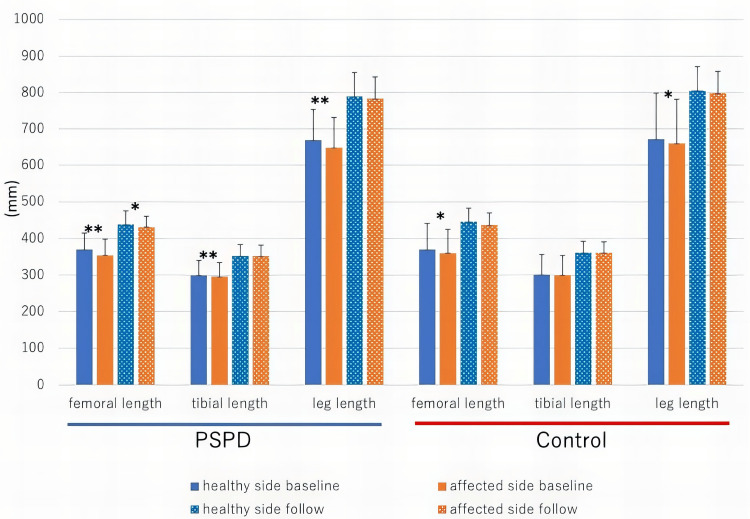
Bone and leg length of the healthy and affected sides at baseline and latest follow-up (mm) *P<0.05. **P<0.01 in Mann–Whitney U test between the sides PSPD: periosteal stripping and periosteal division

Figure 2 shows the bone and leg length of the PSPD and control groups. At baseline, there were significant differences in the femoral and entire leg lengths between the affected and healthy sides in the PSPD (p = 0.002, 0.002, respectively) and control groups (p = 0.031, 0.031, respectively). There was a significant difference in tibial length in the PSPD group (p = 0.007). At the time of the latest follow-up, a difference in femoral length was still noted in the PSPD group (p = 0.049), but there were no significant differences in the entire leg length in the two groups.

Table 2 shows the amount of change in bone length. In the PSPD group, the change in the femoral and tibial lengths of the affected side was significantly greater than that of the healthy side, and the leg length also showed a significantly greater change in the affected side (p = 0.004, 0.043, 0.002, respectively). In the control group, the affected side also tended to show greater changes than the healthy side, but only the tibia length showed a significant difference in the amount of change (p = 0.047).

**Table 2:** Increase in bone length *P<0.05 between the sides in the Wilcoxon signed-rank test. Values are presented in mm, mean ± SD PSPD: periosteal stripping and periosteal division; SD: standard deviation

Group	Increase in femoral length		Increase in tibial length		Increase in whole leg length	
	Healthy side	Affected side	Healthy side	Affected side	Healthy side	Affected side
PSPD	68.8 ± 31.5	77.3 ± 34.9*	53.1 ± 5.5	56.7 ± 26.2*	121.2 ± 55.7	135.1 ± 58.5*
Controls	76.2 ± 49.5	77.3 ± 50.1	59.5 ± 40.1	61.7 ± 40.4*	134.3 ± 89.5	137.5 ± 91.1

Table 3 shows the difference in bone length between the affected and healthy sides. The baseline LLD was 20.5 ± 4.6 mm in the PSPD group, which was significantly greater than that of the control group (11.5 ± 10.0 mm, p = 0.034). However, in the PSPD group, the bone length difference was reduced by 8.5 ± 8.5 and 3.6 ± 5.4 mm in the femur and tibia, respectively, resulting in an LLD of 6.6 ± 10.0 mm at the final observation, and the effect of PSPD on ΔLLD was 13.9 mm. The ΔLLD of PSPD was significantly greater than that of the control group (3.2 ± 8.1 mm, p = 0.034). There was no significant difference in the LLD between the two groups at the final observation.

**Table 3:** Difference in bone length between the affected and healthy side *P<0.05 between PSPD and controls in the Mann–Whitney U test. Values are presented in mm, mean ± SD PSPD: periosteal stripping and periosteal division; SD: standard deviation

Group	Difference in femoral length			Difference in tibial length			Difference in leg length		
	Baseline	Follow-up	Change	Baseline	Follow-up	Change	Baseline	Follow-up	Change
PSPD	15.4 ± 3.0	6.9 ± 10.3	8.5 ± 8.5	4.1 ± 3.8	0.5 ± 5.7	3.6 ± 5.4	20.5 ± 4.6*	6.6 ± 10.0	13.9 ± 7.6*
Controls	9.7 ± 7.2	8.5 ± 10.9	1.2 ± 6.8	2.3 ± 3.1	0.2 ± 3.5	2.2 ± 2.1	11.5 ± 10.0	8.3 ± 13.8	3.2 ± 8.1

**Figure 3:**
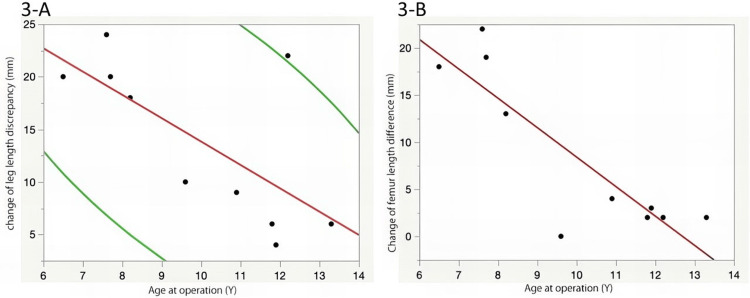
A. Correlation between the age at the time of surgery and change in the leg length discrepancy. B. Correlation between the age at the time of surgery and change in the femoral length difference

The PSPD group showed a significant LLD correction effect, but the effect varied in each case (range: 4-24 mm). There was no correlation between the amount of change in LLD and the follow-up period (p = 0.53); however, a significant correlation was observed between the amount of change in LLD and age at the time of surgery [r = −0.69, 95% confidence interval (CI): −0.92 to −0.1; p = 0.03, Figure 3A]. Especially, there was a strong correlation between the change in femoral length difference and age at the time of surgery (r = −0.87, 95% CI: −0.97 to −0.52, p = 0.0012, Figure 3B). Cases P1, P6, and P7 were operated on at the age of ≤7 years, and there was a reversal of the LLD, with the affected side being longer at the last observation. An LLD correction effect of 22 mm was obtained in patients who underwent PSPD on both the femur and lower leg at the age of 12 years. However, there was no significant correlation between the extent of PSPD performed on the femur (1/3 or 2/3) and the LLD correction effect (p = 0.38). In contrast, the change in LLD in the control group varied from −7 to 14 mm, but there was no significant correlation observed with the follow-up period or age at baseline (p = 0.83 and p = 0.32, respectively).

In the PSPD group, the LLD correction effect of ≥10 mm was obtained in six cases. A logistic regression analysis was performed to determine whether the ΔLLD effect of ≥10 mm could be obtained by the age at the time of surgery. It revealed that age at the time of surgery was a significant factor (p = 0.009, odds ratio: 3.32), and the cutoff value by the receiver operating characteristic curve was 9.6 years (sensitivity: 0.83; specificity: 0.83).

**Figure 4:**
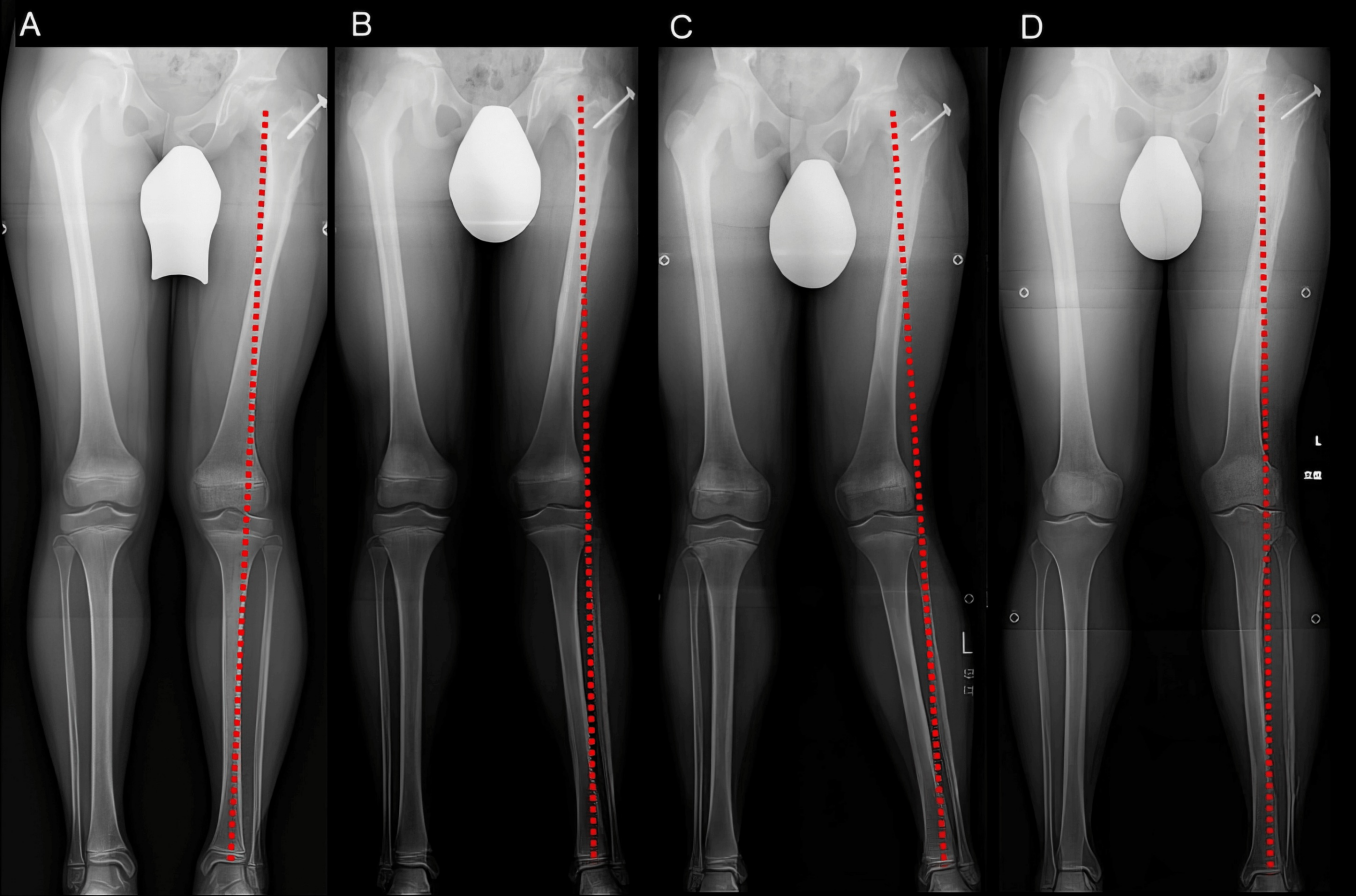
Whole-leg radiographs of a patient who showed a transient genu valgum A: Before PSPD. B: One year after PSPD. C: Two years after PSPD. D: Three years after PSPD PSPD: periosteal stripping and periosteal division

There were no complications related to PSPD, such as vascular or nerve injury, infection, or fracture. During the course after PSPD, five patients had temporary genu valgum of the coronal plane on the affected side (cases 1, 2, 3, 5, and 7). Thereafter, the alignment gradually improved, and the alignment at the final observation showed improvement without significant differences between the healthy and affected sides in all the patients (Figure 4). Similarly, two patients showed a varus knee deformity (cases 4 and 9); however, both cases showed improvement at the final observation.

## Discussion

This study revealed the efficacy and safety of PSPD in improving LLD after proximal femoral intertrochanteric osteotomy for Perthes disease in children. In the control group, a spontaneous correction of LLD was also observed, but the correction was only approximately 28% of the baseline LLD. In contrast, the PSPD group showed a mean correction of 13.9 mm, which was 68% of the baseline LLD, suggesting that the effect of surgery was significant. Additionally, the amount of leg length correction in PSPD was related to the age at the time of surgery. The younger the age at the time of surgery, the greater the LLD correction, and it is more likely that a leg length correction of >10 mm can be achieved if the procedure is performed in patients <10 years of age.

The causes of the overgrowth of the long bone due to PSPD include changes in the hemodynamics and the external force applied to the growth plate. Yabsley et al. reported that periosteal stripping caused a blockage of blood flow to the cortical bone and increased blood flow to the growth plate [24]. Warrell et al. suggested that the development of the growth plate is mechanically controlled by the tension of the periosteum at both ends and that a transverse incision of the periosteum reduces the tension of the periosteum on the growth plate, leading to overgrowth [25].

Regarding the complications associated with PSPD, Sola et al. have reported pathological fractures in animal studies in 5/22 dogs (22%) and 3/10 monkeys (30%) after two repetitions of periosteal stripping [26]. Limpaphayom et al. used a cast for postoperative therapy and allowed weight-bearing walking after a two-week unloading period [18]. In our study, the patient was allowed to walk with half-load from the day after the surgery, and full-load walking was gradually allowed according to the pain intensity; however, none had pathological fractures. Houghton et al. have reported a case of valgus tibia deformity observed when a semi-circumferential periosteal division on the medial side of the tibia was performed [27].

**Figure 5:**
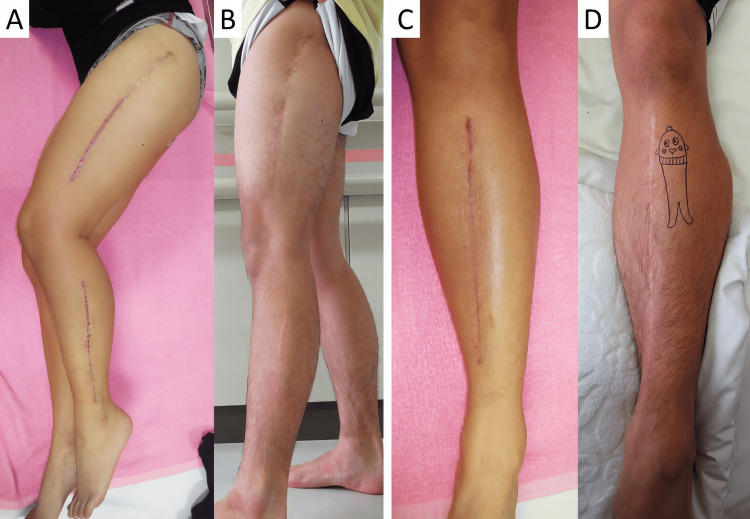
Postoperative scars after PSPD A and C: Scars of the femur, fibula, and tibia at the early postoperative period. B and D: Improved scars at the follow-up six years after the operation

In our study, a transient valgus and varus knee joint deformity was observed in five cases after PSPD. In all cases, the leg alignment improved gradually. An imbalance of periosteal stripping between the medial and lateral sides of the bone may cause these temporal valgus or varus deformities, although the precise mechanism remains unknown. Other complications, such as superficial infection and hematoma, have been reported [28,29]. As for the scar, the wound is long because it covers the entire length of each bone. In the present study, all patients in the surgical group were male, and none of them were concerned about the appearance of the wound at the time of the final observation (Figure 5). However, a careful explanation of the postoperative scar of PSPD before the surgery is necessary.

Regarding the relationship between the age at the time of PSPD and the effect of the bone growth, Limpaphayom et al. retrospectively reviewed 11 patients who underwent PSPD and reported that the mean age at the time of PSPD was 9 ± 2.5 years (range: 5-13 years) [19]. Our study revealed a significant correlation between the age at the time of surgery and ΔLLD. Additionally, logistic regression analysis showed that a >10-mm correction is expected if the patients are <10 years of age at the time of surgery. In contrast, no correlation was confirmed between ΔLLD and follow-up length after the surgery. It is suggested that the age at the time of surgery of the patients, rather than the duration after the surgery, has a greater influence on the amount of leg length correction. No correlation was confirmed between ΔLLD and the extent of surgery (1/3 or 2/3 of femur) either. Nevertheless, one patient aged 12 years obtained ΔLLD of 22 mm after PSPD was performed on the entire femur, tibia, and fibula. This result suggested that, even at an older age, the wide extent of the PSPD is likely more effective. If PSPD was performed on the lower leg as well, the effect on ΔLLD can be expected to be significant. As it is difficult to accurately predict the effect of leg length correction, patients should be thoroughly followed up until their bone has completely matured.

There are several limitations to this study. Firstly, our sample size was relatively small. The cohort was limited to patients who had undergone PSPD for LLD after proximal femoral intertrochanteric osteotomy for Perthes disease, to ensure that patients had similar backgrounds. Clinical studies involving more cases and other diseases are desirable. Secondly, the control group was not randomly selected and included those who had LLD after proximal femoral intertrochanteric osteotomy for Perthes disease but who did not undergo PSPD as per the request of patients or the LLD <20 mm. Given that the patient background, including LLD at baseline, was different from that of the PSPD group, the results were not strictly comparable. The present investigation is a level 4 case series study, and the purpose of having a control group is to show how much LLD is corrected in the natural course. Third, the extent of the surgery varied from patient to patient. One patient who had undergone PSPD of both the femur and lower leg was relatively old, but a leg length correction effect of 22 mm was obtained. However, the effect of the difference in the extent of PSPD performed on the femur was not as strong as the effect of age at the time of surgery on the leg length correction. We recommend further research focusing on the extent of PSPD to the entire leg.

## Conclusions

Based on our findings, PSPD provided a mean leg length correction of 13.9 mm at a mean follow-up duration of 4.3 years in patients with LLD after proximal femoral intertrochanteric osteotomy for Perthes disease. There was a significant correlation between the amount of change in LLD and age at the time of surgery. Obtaining >10 mm limb length correction is more likely if the patients are <10 years of age. No serious adverse effects were reported. PSPD is acceptable in patients because it allows leg-length correction on the affected leg. Although it is difficult to accurately predict the effect of leg length correction, PSPD is a good adjunctive procedure when performing an implant removal surgery for LLD associated with Perthes disease.
